# Wild‐Type Transthyretin Amyloidosis Complicated by Alveolar Hypoventilation due to Diaphragmatic Dysfunction

**DOI:** 10.1002/rcr2.70422

**Published:** 2025-11-29

**Authors:** Mayumi Aoyama, Ryo Takezawa, Rino Arai, Saya Hattori, Takayuki Nakano, Masatsugu Nakano, Motoki Sano, Hidenobu Shigemitsu, Ichiro Kuwahira

**Affiliations:** ^1^ Respiratory Disease Center Tokyo General Hospital Tokyo Japan; ^2^ Department of Cardiology Tokyo General Hospital Tokyo Japan; ^3^ Department of Neurology Tokyo General Hospital Tokyo Japan; ^4^ Department of Critical Care Medicine St. Rose Dominican Siena Hospital Las Vegas Nevada USA

**Keywords:** alveolar hypoventilation, diaphragmatic dysfunction, restrictive pulmonary disease, transthyretin amyloidosis, ultrasound

## Abstract

We report a case of wild‐type transthyretin amyloidosis (ATTRwt) in a 91‐year‐old female who developed alveolar hypoventilation despite improvement in heart failure. The patient presented with dyspnea and lower extremity edema, and was diagnosed with heart failure. ^99^ᵐTc‐pyrophosphate scintigraphy scan showed cardiac uptake consistent with transthyretin amyloidosis, and genetic testing confirmed wild‐type disease. While cardiac symptoms improved with treatment, hypercapnia persisted, prompting pulmonology consultation. Chest radiographs and dynamic magnetic resonance imaging during inspiration and expiration revealed impaired diaphragmatic movement. A chest computed tomography scan showed no significant abnormal findings in the lung fields. Pulmonary function tests revealed mixed ventilatory impairment with preserved total lung capacity but increased residual volume. Maximum inspiratory and expiratory pressures were significantly decreased. Ultrasound evaluation revealed diaphragmatic weakness with minimal thickening during inspiration. Phrenic nerve conduction studies were normal. This case represents the first report of alveolar hypoventilation due to diaphragmatic dysfunction in ATTRwt.

## Graphical abstract

We report the first case of alveolar hypoventilation due to diaphragmatic dysfunction in ATTRwt. Chest radiographs show little change in the height of the diaphragm during expiration (A) and inspiration (B). Ultrasound evaluation shows minimal diaphragmatic thickening during inspiration.

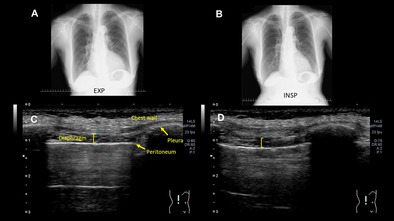

## 1 Introduction

Wild‐type transthyretin amyloidosis (ATTRwt) is an increasingly recognised cause of heart failure in elderly patients, characterised by amyloid fibril deposition in cardiac tissue [1]. While cardiac manifestations are well established, respiratory complications are rarely reported. We present the first case of ATTRwt complicated by alveolar hypoventilation due to diaphragmatic dysfunction, demonstrating an uncommon but clinically significant respiratory manifestation of this systemic disease.

## 2 Case Report

A 91‐year‐old female presented with progressive dyspnea and bilateral lower extremity edema. Her medical history included glaucoma, cataract, and duodenal ulcer. Family history was significant for cerebral infarction (father) and gastric cancer (mother, brother). She had a 20 pack‐year smoking history. She was admitted to the cardiology department for further evaluation.

**FIGURE 1:**
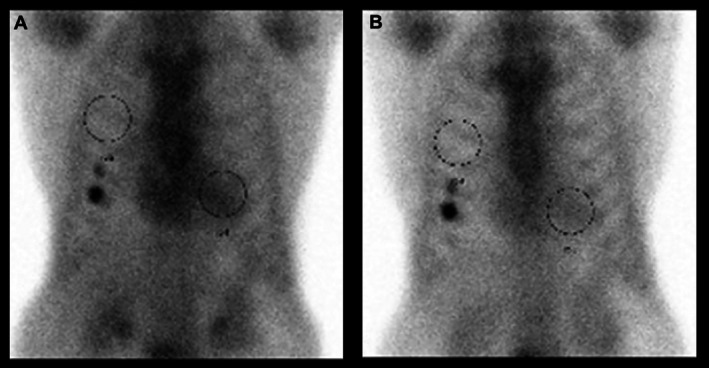
^99^ᵐTc‐pyrophosphate scintigraphy scan (A, B). A is the scan result after 1 h, and B is after 3 h. ^99^ᵐTc‐pyrophosphate scintigraphy scan showed Grade 2 cardiac uptake with heart‐to‐contralateral lung ratio of 1.54 (A) and 1.44 (B), consistent with transthyretin cardiac amyloidosis. The accumulation in the lower right rib cage is due to a past fracture.

Cardiac evaluation revealed severely reduced left ventricular ejection fraction of 23.7% on echocardiography with global hypokinesis. Electrocardiography showed sinus rhythm with low voltage. ^99^ᵐTc‐pyrophosphate scintigraphy scan showed Grade 2 cardiac uptake with heart‐to‐contralateral lung ratio of 1.54 (1 h) and 1.44 (3 h), consistent with transthyretin cardiac amyloidosis (Figure 1A,B). Delayed enhancement magnetic resonance imaging showed negative late gadolinium enhancement with positive dark blood pool sign. Coronary angiography demonstrated no significant stenosis. While cardiac symptoms gradually improved with Guideline‐Directed Medical Therapy, hypercapnia persisted, prompting pulmonology consultation.

The patient is 155 cm tall and weighs 51 kg corresponding to a body mass index (BMI) of 21.2 kg/m^2^. Physical examination revealed no abnormalities in vital signs with oxygen saturation of 92% on room air. Cardiovascular examination showed regular heart sounds without murmurs, and pulmonary examination revealed clear breath sounds bilaterally. Neurological examination demonstrated diminished deep tendon reflexes, particularly absent bilateral Achilles reflexes and reduced patellar reflexes, without macroglossia or carpal tunnel syndrome.

Laboratory studies showed mild anaemia (haemoglobin 10.6 g/dL), elevated B‐type natriuretic peptide (34.8 pg/mL), and normal inflammatory markers. Arterial blood gas analysis on room air obtained in the supine position revealed hypoxemia and hypercapnia (pH 7.396, PaCO_2_ 57.5 mmHg, PaO_2_ 64.5 mmHg, HCO_3_^−^ 34.5 mmol/L). The alveolar–arterial oxygen pressure difference was normal at 14 mmHg. Based on the results of arterial blood gas analysis, the cause of hypoxemia was considered solely due to alveolar hypoventilation. Genetic analysis for transthyretin mutations was negative, confirming ATTRwt. Serum amyloid A protein was normal, and immunoelectrophoresis was negative for M protein and Bence Jones protein.

**FIGURE 2:**
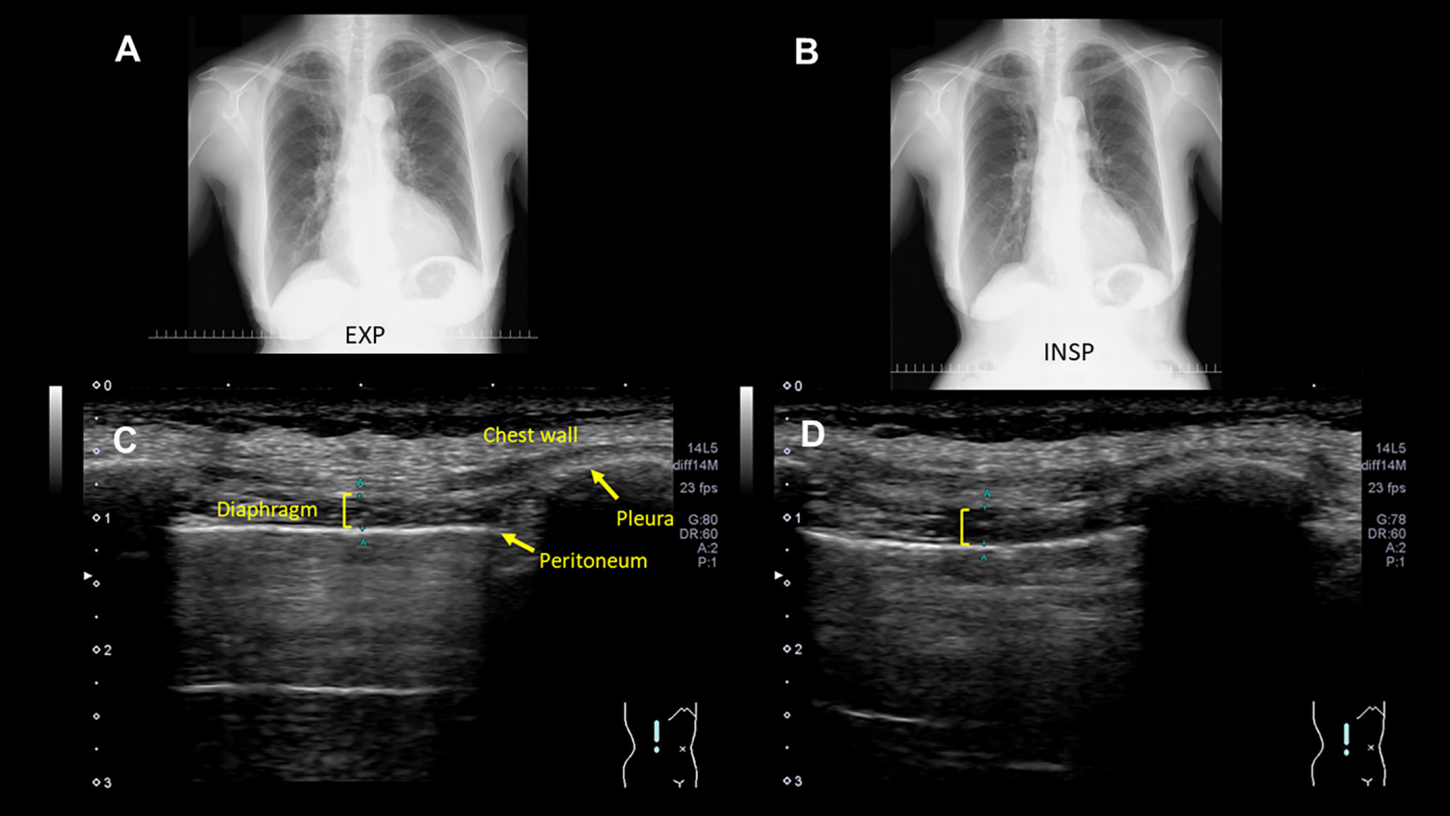
Chest radiographs during maximum expiration (A) and inspiration (B) and diaphragmatic ultrasound assessment (C). Chest radiographs show little change in the height of the diaphragm during maximum expiration (A) and inspiration (B). Ultrasound evaluation demonstrating minimal diaphragmatic thickening during maximum inspiration. Left panel (C) shows the thickness was 2.5 mm at end‐expiration (TDE) and right panel (D) shows it was 2.8 mm at end‐inspiration (TDI). This represents a thickening fraction (TDF) of 12% ((TDI − TDE)/TDE × 100), well below the normal threshold of 20%, indicating diaphragmatic dysfunction.

Chest radiographs during maximum expiration and inspiration revealed impaired diaphragmatic movement (Figure 2A,B). Little change in the height of the diaphragm was observed during expiration. Chest computed tomography scan showed no significant abnormal findings in the lung fields. Dynamic magnetic resonance imaging during deep inspiration and expiration also showed markedly impaired diaphragmatic movement (Video not shown).

Pulmonary function testing demonstrated severe restrictive and obstructive impairment with a vital capacity of 1.03 L (48.1% predicted), forced vital capacity of 0.90 L (42.1% predicted), and forced expiratory volume in 1 s of 0.41 L (24.0% predicted). Notably, total lung capacity was preserved at 3.64 L (101.4% predicted) with markedly increased residual volume of 2.61 L (138.8% predicted), resulting in an elevated residual volume/total lung capacity ratio of 71.7%. Diffusing capacity of the lung for carbon monoxide was 10.23 mL/min/mmHg (80.0% predicted). Respiratory muscle strength was severely impaired with maximum expiratory pressure of 25 cm H_2_O and maximum inspiratory pressure of 35 cm H_2_O (reference values for Japanese women: 42 and 60 cm H_2_O, respectively [2, 3]). Diaphragmatic ultrasound evaluation demonstrated significant dysfunction with minimal thickening during maximum inspiration. The diaphragm thickness at end‐expiration (TDE) was 2.5 mm, and at end‐inspiration (TDI) increased only to 2.8 mm, representing a thickening fraction (TDF) of 12% ((TDI − TDE)/TDE × 100), well below the normal threshold of 20% (Figure 2C,D) [4].

Peripheral nerve conduction studies revealed reduced motor nerve conduction velocities in multiple nerves (bilateral median nerves, tibial nerves, and peroneal nerves) while sensory conduction remained largely normal. However, diaphragmatic evoked potentials showed normal latencies (left: 8.3 ms, right: 10.5 ms) and amplitudes (left: 721.7 μV, right: 789.4 μV), indicating that phrenic nerve conduction was normal. To differentiate neuromuscular disorders such as myasthenia gravis, muscular dystrophy, Guillain‐Barré syndrome, chronic inflammatory demyelinating polyneuropathy and other related conditions, we performed electromyography (EMG) and cerebrospinal fluid (CSF) analysis in addition to peripheral nerve conduction studies. Based on the comprehensive evaluation above, no findings suggestive of other neuromuscular disorders were identified. Furthermore, handgrip strength was 17 kg on the right and 13 kg on the left, which was below the cutoff value for women (< 18 kg) proposed by the Asian Working Group for Sarcopenia [5]. On manual muscle testing (MMT), hip flexion was grade 4 and knee extension was grade 5 bilaterally. Although the handgrip strength was slightly reduced, lower limb strength and daily activity were well preserved. The findings were interpreted as age‐appropriate physiological decline rather than pathological sarcopenia.

Since desaturation was observed during the night, sleep apnea screening was performed using a portable monitoring device, which recorded nasal airflow, oxygen saturation, and heart rate. The measurement results showed an average apnea‐hypopnea index of 19 events per hour. To prevent worsening hypoxemia and hypercapnia during sleep, we recommended continuous positive airway pressure (CPAP) therapy or noninvasive positive pressure ventilation (NPPV) to the patient. However, she declined CPAP therapy and NPPV. Consequently, we decided to initiate nocturnal home oxygen therapy at 0.25 L/min to prevent the risk of worsening hypercapnia as a minimum alternative.

Since the patient was clinically stable, she was discharged and followed up at the outpatient department. She declined high‐cost medications such as Tafamidis and preferred Guideline‐Directed Medical Therapy. Bronchodilator treatment was also added.

## 3 Discussion

This case represents a unique manifestation of ATTRwt presenting with persistent alveolar hypoventilation due to diaphragmatic dysfunction. According to the guideline [1], the diagnosis of transthyretin amyloidosis (ATTR) was established through positive ^99^ᵐTc‐pyrophosphate scintigraphy scan with exclusion of light chain amyloidosis, which has been reported to have 100% specificity for ATTR cardiac amyloidosis [1, 6].

The mechanism of diaphragmatic dysfunction in this case appears to be mechanical restriction due to amyloid fibril deposition rather than phrenic nerve involvement, as evidenced by normal phrenic nerve conduction studies and the absence of diaphragmatic elevation. Dynamic magnetic resonance imaging also showed markedly impaired diaphragmatic movement. The preserved total lung capacity with increased residual volume and reduced respiratory muscle strength supports this hypothesis. Since BMI was 21.2 kg/m^2^, which represents a standard typical body habitus for Japanese women, obesity hypoventilation syndrome was not likely. Furthermore, EMG and CSF analysis showed no findings suggestive of other neuromuscular disorders.

While diaphragmatic involvement has been reported in six cases of light chain amyloidosis [7], respiratory muscle involvement in ATTR is extremely rare, with only one previous case of phrenic nerve palsy reported [8]. Invasive examinations such as diaphragmatic biopsy could not be performed in this case because the patient declined invasive procedures. The mechanism by which amyloid fibres deposited in the diaphragm and restricted its movement can be inferred from the clinical findings. The reason why the phrenic nerve was not affected in this case is unclear. Although the term “amyloid myopathy” may be appropriate in addition to cardiomyopathy, the handgrip strength and lower limb strength assessed by MMT were maintained at an age‐appropriate level, indicating weakness of other muscles was not present. It has been reported that abnormalities may be detected on peripheral nerve conduction studies even in the absence of subjective symptoms such as muscle weakness [9, 10]. In this case, the impairment of the diaphragm being more pronounced compared to other muscles except for the myocardium may be characteristic. Our case appears to be the first report of alveolar hypoventilation due to diaphragmatic dysfunction in ATTRwt.

This case emphasises the importance of comprehensive respiratory assessment, including arterial blood gas analysis, pulmonary function testing and diaphragmatic function evaluation, in amyloidosis patients presenting with reduced oxygen saturation, particularly when standard cardiac treatment fails to resolve hypercapnia and hypoxemia.

## Author Contributions

**M.A., R.T., R.A., S.H., T.N,** and **I.K.:** conception or design of the work, the acquisition, analysis or interpretation of data for the work. **M.A, M.N., M.S., H.S.,** and **I.K.:** drafting the work or reviewing it critically for important intellectual content. **M.A., R.T., R.A., S.H., T.N., M.N., M.S., H.S.,** and **I.K.:** final approval of the version to be published.

## Funding

The authors have nothing to report.

## Consent

The authors declare that written informed consent was obtained for the publication of this manuscript and accompanying images using the consent form provided by the Journal.

## Conflicts of Interest

The authors declare no conflicts of interest.

## Data Availability

The data that support the findings of this study are available on request from the corresponding author. The data are not publicly available due to privacy or ethical restrictions.

## References

[1] H. Kitaoka , C. Izumi , Y. Izumiya , et al., “JCS 2020 Guideline on Diagnosis and Treatment of Cardiac Amyloidosis,” Circulation Journal 84 (2020): 1610–1671. 32830187 10.1253/circj.CJ-20-0110

[2] M. Suzuki , S. Teramoto , E. Sudo , et al., “Age‐Related Changes in Static Maximal Inspiratory and Expiratory Pressures” [in Japanese], Nihon Kyōbu Shikkan Gakkai Zasshi 35 (1997): 1305e11. 9567073

[3] R. Hamada , Y. Oshima , Y. Yoshioka , et al., “Comparison of International and Japanese Predictive Equations for Maximal Respiratory Mouth Pressures,” Respiratory Investigation 60 (2022): 847–851. 36038474 10.1016/j.resinv.2022.07.003

[4] E. Gottesman and F. D. McCool , “Ultrasound Evaluation of the Paralyzed Diaphragm,” American Journal of Respiratory and Critical Care Medicine 155 (1997): 1570–1574. 9154859 10.1164/ajrccm.155.5.9154859

[5] L. K. Chen , J. Woo , P. Assantachai , et al., “Asian Working Group for Sarcopenia: 2019 Consensus Update on Sarcopenia Diagnosis and Treatment,” Journal of the American Medical Directors Association 21 (2020): 300–307. 32033882 10.1016/j.jamda.2019.12.012

[6] S. Bokhari , A. Castaño , T. Pozniakoff , S. Deslisle , F. Latif , and M. S. Maurer , “ ^99m^Tc‐Pyrophosphate Scintigraphy for Differentiating Light‐Chain Cardiac Amyloidosis From the Transthyretin‐Related Familial and Senile Cardiac Amyloidoses,” Circulation. Cardiovascular Imaging 6 (2013): 195–201. 23400849 10.1161/CIRCIMAGING.112.000132 PMC3727049

[7] K. M. Moffitt and L. Lin , “A Case of Fatal Respiratory Failure Caused by Diaphragmatic Amyloidosis,” American Journal of Respiratory and Critical Care Medicine 199 (2019): A1773.

[8] T. Sekiguchi , H. Tomimitsu , Y. Nishida , T. Irioka , A. Inaba , and Y. Hoshii , “ATTR Amyloidosis Complicated by Phrenic Nerve Palsy,” Canadian Journal of Neurological Sciences 40 (2013): 129–130. 10.1017/s0317167100017431 23427361

[9] D. Namiranian and S. Geisler , “Neuromuscular Complications of Systemic Amyloidosis,” American Journal of Medicine 135 (2022): S13–S19. 35104443 10.1016/j.amjmed.2022.01.006

[10] L. F. Pinto and M. V. Pinto , “Neuromuscular Amyloidosis,” Practical Neurology 21 (2021): 63–68.

